# Differential Antibody Response to mRNA COVID-19 Vaccines in Healthy Subjects

**DOI:** 10.1128/spectrum.00341-21

**Published:** 2021-08-04

**Authors:** Sarah E. Wheeler, Galina V. Shurin, Mary Yost, Adam Anderson, Lisa Pinto, Alan Wells, Michael R. Shurin

**Affiliations:** a Department of Pathology, University of Pittsburgh grid.471408.egrid.21925.3d Medical Center, Pittsburgh, Pennsylvania, USA; b Bio-Rad Laboratories, Inc., Benicia, California, USA; c Department of Immunology, University of Pittsburgh grid.471408.egrid.21925.3d Medical Center, Pittsburgh, Pennsylvania, USA; Fundacio irsiCaixa

**Keywords:** antibody, COVID-19, SARS-CoV-2, vaccination, humoral immunity

## Abstract

Knowledge about development and duration of virus-specific antibodies after COVID-19 vaccination is important for understanding how to limit the pandemic via vaccination in different populations and societies. However, the clinical utility of postvaccination testing of antibody response and selection of targeted SARS-CoV-2 antigen(s) has not been established. The results of such testing from clinical teams independent from vaccine manufacturers are also limited. Here, we report the initial results of an ongoing clinical study on evaluation of antibody response to four different SARS-CoV-2 antigens after first and second dose of Pfizer and Moderna mRNA vaccines and at later time points. We revealed a peak of antibody induction after the vaccine boosting dose with a gradual decline of antibody levels at later time. Anti-nucleocapsid antibody was not induced by spike protein-encoding vaccines and this may continue to serve as a marker of previous SARS-CoV-2 infection. No differences between the two vaccines in terms of antibody response were revealed. Age and gender dependencies were determined to be minimal within the healthy adult (but not aged) population. Our results suggest that postvaccination testing of antibody response is an important and feasible tool for following people after vaccination and selecting individuals who might require a third dose of vaccine at an earlier time point or persons who may not need a second dose due to previous SARS-CoV-2 infection.

**IMPORTANCE** Now that authorized vaccines for COVID-19 have been widely used, it is important to understand how they induce antivirus antibodies, which antigens are targeted, how long antibodies circulate, and how personal health conditions and age may affect this humoral immunity. Here, we report induction and time course of multiple anti-SARS-CoV-2 antibody responses in healthy individuals immunized with Pfizer and Moderna mRNA vaccines. We also determined the age and gender dependence of the antibody response and compared antibody levels to responses seen in those who have recovered from COVID-19. Our results suggest the importance of screening for antibody response to multiple antigens after vaccination in order to reveal individuals who require early and late additional boosting and those who may not need second dose due to prior SARS-CoV-2 infection.

## INTRODUCTION

Severe acute respiratory syndrome coronavirus 2, SARS-CoV-2, is a single-stranded RNA virus which can be passed between humans and is the cause of the coronavirus pandemic, which began in 2019. Infection with SARS-CoV-2 may result in coronavirus disease 2019 (COVID-19), which commonly induces a robust and persistent immune response to SARS-CoV-2. This includes virus-specific antibodies, memory B cells, and effector and memory CD8^+^ T cells (1, 2). The U.S. Food and Drug Administration (FDA) issued an Emergency Use Authorization (EUA) for the first two SARS-CoV-2 vaccines in December 2020—the Pfizer-BioNTech and Moderna vaccines—for the prevention of COVID-19 in the United States and the protection of persons who are at high risk for complications (3, 4). Both vaccines use nucleoside-modified mRNA with a lipid nanoparticle-formulation to encode the spike (S) protein of SARS-CoV-2.

It is considered critical that SARS-CoV-2 vaccines provoke a strong immune response against the spike protein, particularly the receptor-binding domain (RBD) of the spike protein which contains many neutralizing epitopes (5). Therefore, many vaccines utilize stabilizing mutations in the S glycoprotein to maintain the prefusion conformation and prevent shedding of the S1 subunit (6). Available information to date seems to indicate that these mutated S proteins are more immunogenic than the wild-type S protein. Both the mRNA-1273 vaccine (Moderna) and the BNT162b2 vaccine (Pfizer) (7) encode the full-length viral S ectodomain with a transmembrane anchor and two S-2P mutations which serve to stabilize the prefusion conformation (7, 8).

Initially, Pfizer clinically investigated the BNT162b1 vaccine candidate that incorporates modified mRNA to encode only the RBD portion of the S protein, thought to be the key target of virus-neutralizing antibodies (9). The RBD antigen that resulted from BNT162b1 had an additional T4 fibritin-derived fold in the trimerization domain which served to increase immunogenicity (10). Later, Pfizer reported that the BNT162b2, which encoded the full-length stabilized S glycoprotein, elicited dose-dependent SARS-CoV-2 neutralizing antibody titers comparable or higher than those elicited by a defined panel of convalescent SARS-CoV-2 serum samples (11). The full-length protein vaccine was shown to have fewer side effects and was better tolerated than other vaccine candidates. Using the full-length glycoprotein vaccine, a randomized, placebo-controlled trial was performed with over 40,000 participants. The trial found that two doses of BNT162b2 demonstrated 95% protection against COVID-19 (7).

The Moderna mRNA-1273 vaccine encodes the S-2P antigen (15-1208; a mutant recombinant version of the S glycoprotein with proline replacements at amino acids 986 and 987) (12, 13), a transmembrane anchor, and an S1-S2 cleavage site. The prefusion conformation is stabilized by the consecutive proline substitutions which are located in the S2 subunit at the top of the central helix (6, 14). Findings from the 30,000-participant phase III clinical trial demonstrated an efficacy of 94.1% after a median follow-up of 2 months in preventing symptomatic COVID-19 with laboratory confirmation of infection. This trial excluded those previously infected with SARS-CoV-2 and was a randomized, double-blind, placebo-controlled trial (15).

The mRNA COVID-19 vaccine series is comprised of two doses administered intramuscularly: 30 μg, 0.3 ml each with 3 weeks apart for Pfizer-BioNTech and 100 μg, 0.5 ml each with 1 month apart for Moderna. Several reports documented a strong humoral response to mRNA vaccines (16–18). These vaccines have shown efficacy against the mutated strains of the virus (19–21). For instance, participants who were fully immunized with the Moderna mRNA-1273 COVID-19 vaccine have sera that demonstrates neutralizing activity against the SARS-CoV-2 variants: the Alpha variant (B.1.1.7), the Beta variant (B.1.351-v1, B.1.351-v2, and B.1.351-v3), the Delta variant (B.1.617.2), and the Gamma variant (P.1) (22). In addition, neutralization titers in vaccinated participants often exceed those of convalescent COVID-19 patients who were not hospitalized (23). Estimation of Pfizer-BioNTech BNT162b2 vaccine effectiveness against mutated variants using a test-negative case-control study design revealed its effectiveness against the B.1.1.7 and B.1.351 variants (23, 24). Pfizer-BioNTech vaccine is also effective against Delta and Kappa variants of the coronavirus (25). Interestingly, SARS-CoV-2 mRNA vaccination also induces cross-reactive antibodies to seasonal β-coronaviruses (26).

Although the immune response against SARS-CoV-2 has been well characterized in naturally infected people, development of immunity after administration of antiviral vaccines, including mRNA vaccines, is not yet completely understood. For instance, simultaneous analysis and comparison of antibodies to four SARS-CoV-2 antigens in healthy volunteers receiving two mRNA vaccines has not yet been conducted. The availability of limited data about humoral response to vaccination may be in part due to the fact that the CDC does not recommend measuring postvaccination titers for COVID-19: “Antibody testing is not currently recommended to assess for immunity to SARS-CoV-2 following COVID-19 vaccination because the clinical utility of postvaccination testing has not been established” (27). However, to reduce the current pandemic and limit the burden of COVID-19 worldwide by effectively vaccinating as many people as possible, we must also select and validate appropriate assays and comprehensively characterize antiviral immunity associated with COVID-19 vaccines.

Although a consistent proof of protective immunity after vaccination may only come via reinfection challenge experiments or longitudinal studies of postvaccination individuals, studies analyzing antibody response to different SARS-CoV-2 antigens after vaccination of healthy volunteers and recovered COVID-19 individuals are important for establishing the “levels of protection” to be determined by FDA-approved clinical laboratory assays. In addition, testing of vulnerable populations for sufficient vaccine response will be critical in stemming the spread in these populations and requires that we define a normal or sufficient vaccine response for comparison. The goal of this study was to determine the level of antibodies to RBD, S1, S2, and nucleocapsid (N) SARS-CoV-2 antigens in the sera of volunteers receiving Pfizer-BioNTech and Moderna vaccines by evaluating the feasibility of novel BioPlex 2200 SARS-CoV-2 IgG panel. Our results demonstrate the dynamic of antibody production after the two-dose vaccine schedule and establish differences between antibody generation induced by a vaccine and natural COVID-19 disease. Although some of the immune mechanisms described here are not novel, our study provides critical, confirming evidence that key antibody pathways are indeed modulated under the COVID-19 vaccination procedure.

## RESULTS

### Dynamics of antibody response to vaccination.

**FIG 1:**
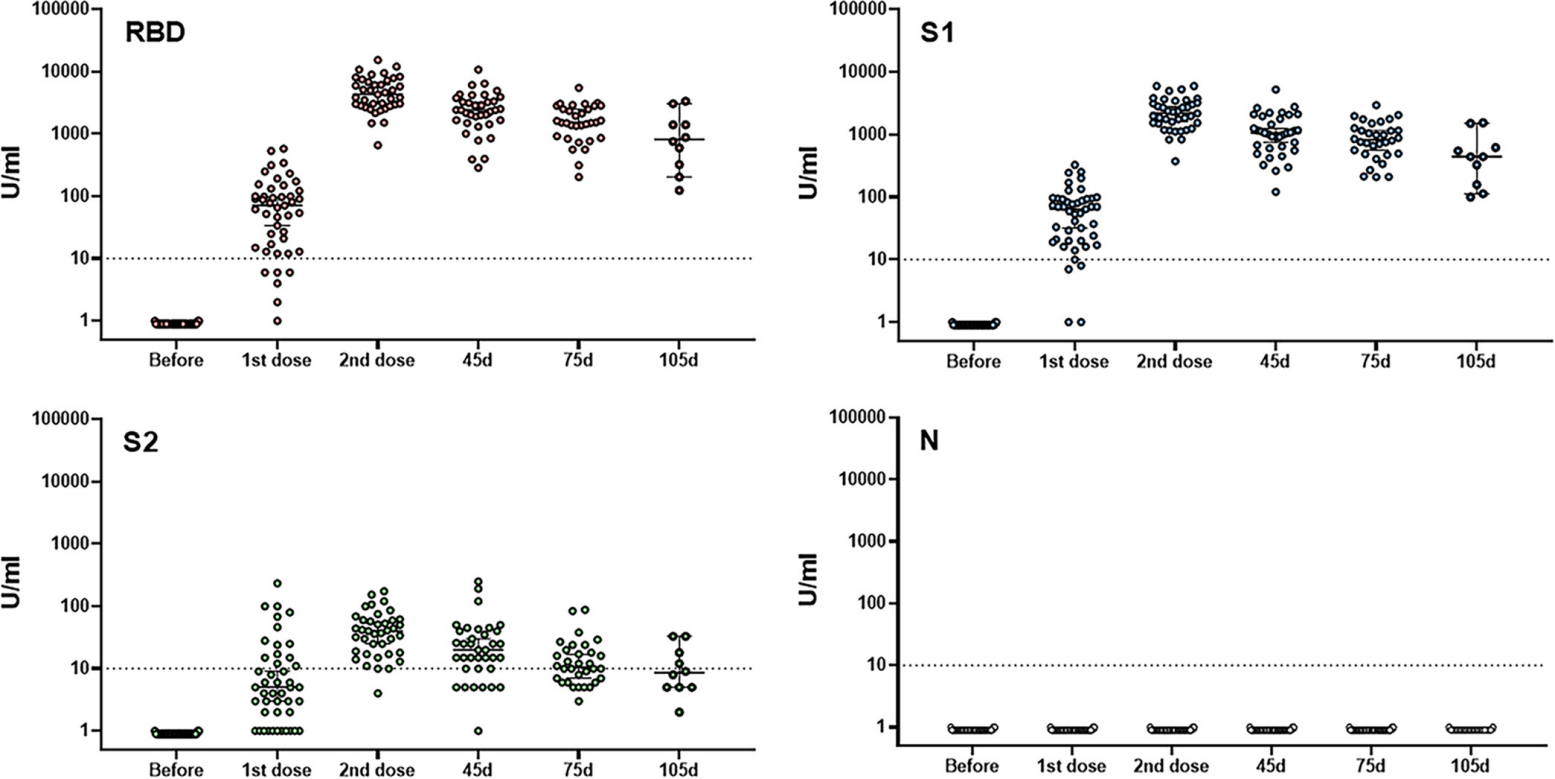
Individual antibody responses to two doses of mRNA vaccines. Blood specimens were collected from healthy individuals before and after the first and second doses of the Pfizer or Moderna vaccines and then every month. IgG antibodies binding to RBD, S1, S2, and nucleocapsid SARS-CoV-2 structural proteins were screened and differentiated by the BioPlex 2200 SARS-CoV-2 IgG multiplex panel as described in Materials and Methods. Each time point represents individual values with the mean and SEM. 10 U/ml is the negative cutoff concentration.

A common measure of the effectiveness of a vaccine is the specific antibody response in serum samples. Common patterns of antibody response to four different SARS-CoV-2 antigens after vaccination of healthy individuals are shown in Fig. 1. All volunteers demonstrated no antibodies to any tested antigens before vaccine administration. The first dose of a vaccine induced the production of specific antibodies within 2 weeks, although the levels varied from negative to 580 (median, 71), 327 (median, 64), and 234 (median, 5) U/ml for RBD, S1, and S2 antibodies, respectively. Interestingly, 13, 3, and 66% of participating volunteers demonstrated negative (<10 U/ml) antibody responses to RBD, S1, and S2 antigens, respectively, after the first dose vaccine administration. No anti-nucleocapsid antibodies were detected during or after vaccination. The second vaccine dose caused a significant upregulation of tested antibodies of up to 100-fold in all volunteers, including statistically significant increases in those who demonstrated negative or low response to first vaccine dose (Fig. 1). The levels of antibodies reached 15,300 (median, 4,320), 5940 (median, 2,172), and 174 (median, 40) U/ml for RBD, S1, and S2, respectively (Fig. 1). No nonresponders were detected after second vaccine dose administration for anti-RBD and -S1 antibodies, and only one person demonstrated no response for anti-S2 antibody (Fig. 1). Interestingly, the levels of anti-RBD and anti-S1 antibodies after the second dose in all participants were higher than any levels seen after the first dose, regardless of the responsiveness to the first dose; this suggests that even if scored as negative, the initial vaccine dose did induce an immunologic response.

**FIG 2:**
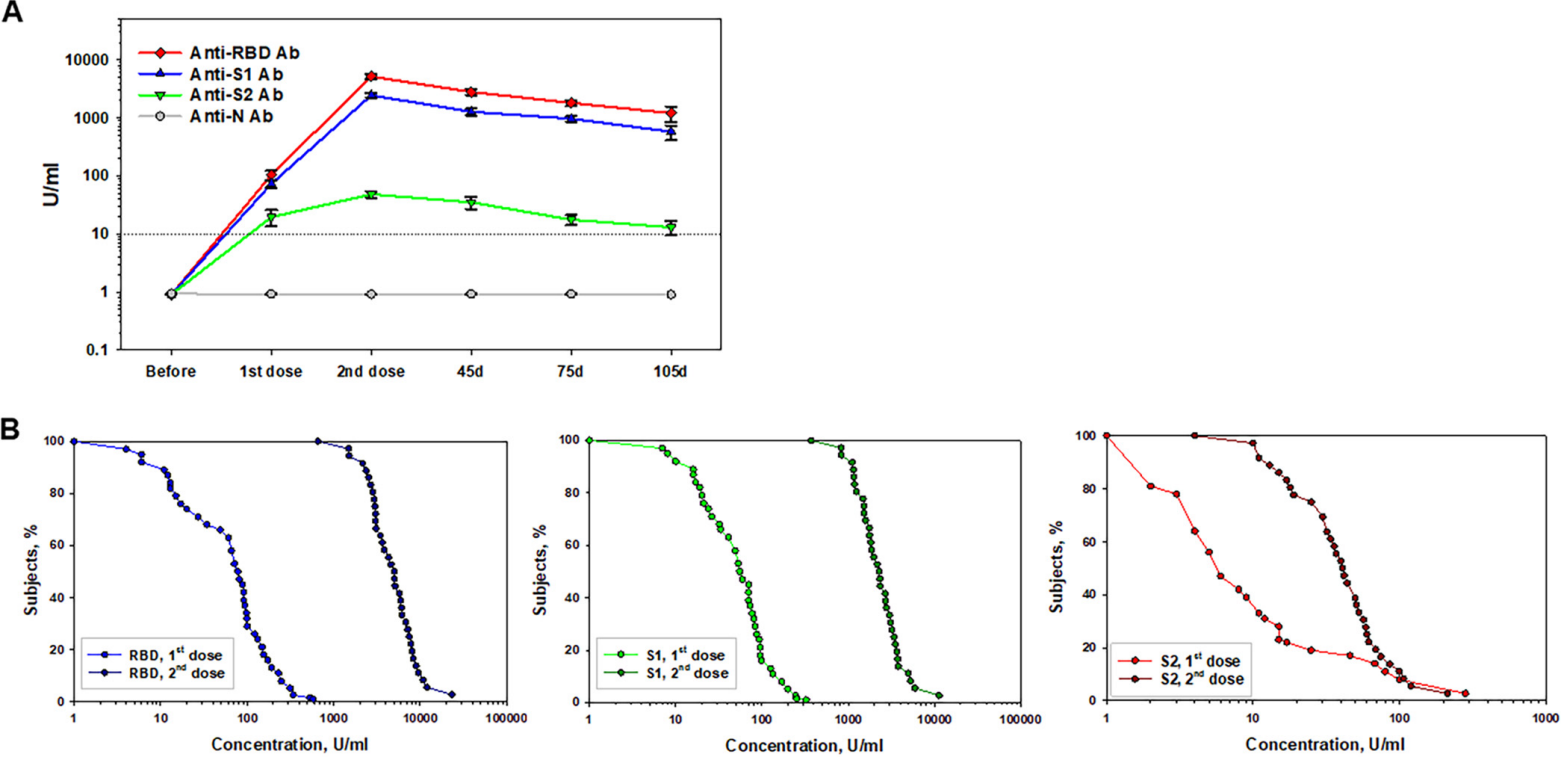
Time-course of antibody response to vaccination with mRNA vaccines. Antibody responses to two doses of Pfizer and Moderna vaccination were assessed for RBD, S1, S2, and nucleocapsid. (A) Summation of two-dose vaccine-induced antibody development to different SARS-CoV-2 antigens in healthy volunteers. The *x* axis shows time points before and after two doses of vaccine administration and 45 and 75 days after a completed vaccination; the *y* axis shows the logarithmic antibody concentrations shown as means ± the SEM. Significant differences with corresponding *P* values for each antigen are presented in the text. 10 U/ml is the negative cutoff concentration. (B) RCDCs of antibody response in healthy volunteers postvaccination for each of the three antigens. The *x* axis represents the antibody concentration values in log scale; the *y* axis is the proportion of subjects having at least that antibody level. The curve begins at 100% and then descends from left to right. When the RCD curves overlap, the two doses of vaccines induced comparable immune responses. If one curve is above another one, it indicates the higher immune response.

The next set of data includes anti-Spike antibody levels after a completed vaccination on a monthly basis. Figure 2A summarizes these results for each antigen. Kruskal-Wallis one-way analysis of variance (ANOVA) on ranks with all pairwise comparisons (Dunn’s method for unequal group sizes) revealed a statistically significant difference in the levels of anti-RBD antibodies (nonlogarithmic values). For instance, the “1st dose” group (median, 71.0) versus the “2nd dose” group (median, 4320.0) (*P* < 0.001) and the “45d” group (median, 2419.0) (*P* < 0.001) and the “75d” group (median, 1514.0) (*P* < 0.001). A decrease in anti-RBD antibody after its peak in “2nd dose” group was not significant at day 45 (*P* = 0.056) but was significant on day 75 (*P* < 0.001). At the same time, the difference between the “45d” and “75d” groups was not statistically significant for anti-RBD antibody levels (*P* = 0.752).

Similar results were obtained for anti-S1 IgG. The “1st dose” group (median, 64.0) data were significantly lower than data obtained from all other groups: the “2nd dose” group (median, 2172.0), the “45d” group (median, 1053.5) and the “75d” group (median, 821.0) (all *P* < 0.001). The decrease in anti-S1 antibody at 45 and 75 days after the second dose was significant (*P* = 0.013 and *P* < 0.001, respectively), although no differences between the “45d” and “75d” groups were seen (*P* = 1.0).

Changes in anti-S2 antibody levels were slightly different. Although a second dose of a vaccine induced a significantly stronger anti-S2 IgG levels (median, 40.0 versus 5.0, *P* < 0.001), the decrease on day 45 after a second shot (median, 20.0) did not reach statistical significance (*P* = 0.121), whereas it was significant on day 75 (median, 10.5, *P* < 0.001). No differences between the “45d” and “75d” groups were detected (*P* = 0.518).

To display the distribution of anti-SARS-CoV-2 antibody values and for visual comparisons of distributions between different time points in vaccine groups, the reverse cumulative distribution curves (RCDCs) are shown in Fig. 2B. In all groups prevaccination, the RCD curves were similar for each of the tested antigens, indicating a lack of baseline serological bias between study groups (data not shown). Postvaccination, the proportion of subjects reaching higher antibody levels to RBD, S1, and S2 proteins increased. For the RBD and S1 antigens, the RCD curves for the second dose of a vaccine are above the curve for the first dose of a vaccine at all antibody concentrations presented in a log scale. This suggests that the vaccine dose with the highest curve induced the greatest immune responses. For the S2 antigen, two RCD curves coincide at the highest antibody levels, which demonstrates comparable immune responses. While the postvaccination RCD curves were similar for RBD and S1 antigens, for the S2 antigen there was a shift in the “2nd dose” RCDC, demonstrating that a lower proportion of subjects achieved antibody concentrations of >100 U/ml versus the “1st dose” group (Fig. 2B).

Together, these data demonstrate that none of volunteers had serologic signs of previous natural SARS-CoV-2 infection, all individuals responded to two-dose mRNA vaccination by a significant production of antibodies specific to spike proteins encoded by mRNA vaccines, and the peak of antibody response was seen 2 weeks after second dose of the vaccine.

### Difference in antibody response to two vaccines.

**FIG 3:**
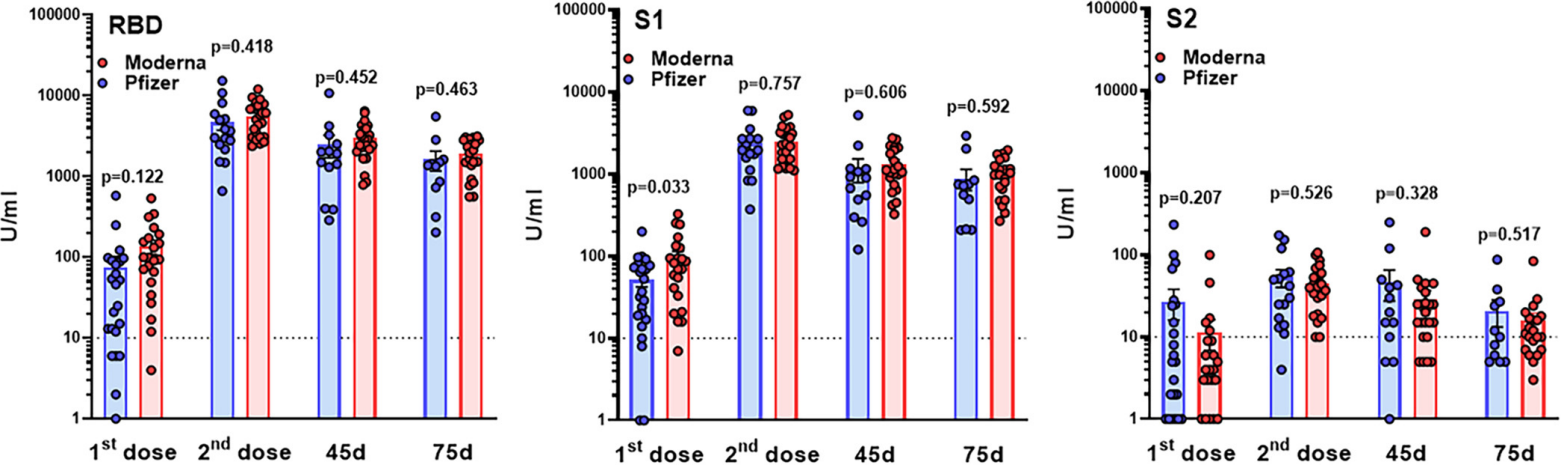
Comparative analysis of antibody response to two mRNA vaccines. Antibody responses to two doses of Pfizer and Moderna vaccination of healthy volunteers were assessed by the BioPlex 2200 SARS-CoV-2 IgG multiplex panel. Results represent individual values (dots) and means ± the SEM (bars) for IgG antibodies recognizing RBD, S1, and S2 SARS-CoV-2 antigens. *P* values were determined using an unpaired *t* test. 10 U/ml (dotted line) is the negative cutoff concentration.

To compare the strength and duration of antibody response to Pfizer and Moderna vaccines, we assessed levels of antibodies to three antigens at different time points in individuals receiving Pfizer (51%; Table 1) or Moderna (49%) vaccines. Analysis of results shown in Fig. 3 utilizing unpaired parametric *t* test with Welch’s correction (after evaluation for normal distribution by Kolmogorov-Smirnov test) revealed no statistically significant differences in the potency of immune response to RBD protein after first and second doses of either mRNA vaccine. No statistically significant differences in the duration of anti-RBD responses to Pfizer and Moderna vaccines were also detected (Fig. 3). Antibody response to S1 protein showed a significantly higher response to first dose of Moderna (96.23 ± 18.13 U/ml) than Pfizer (51.61 ± 9.54) vaccine, while no statistically significant differences were detected at all following time points. Finally, no differences between two vaccines in antibody response to S2 protein after vaccination were determined (Fig. 3). Overall, we did not reveal differences between Pfizer and Moderna in terms of the antibody response to spike protein after two-dose vaccination in healthy volunteers.

**TABLE 1:** Demographic data of participated individuals

Characteristic	Data
Total no. of participants	47
	
Age (yr)	
Median	50.00
Mean ± the SD	49.29 ± 12.81
Range	19–70
	
No. (%) of subjects	
Female	33 (70)
Pfizer	24 (51)
Female	16 (67)
Moderna	23 (49)
Female	16 (70)
Occupational risk	32 (68)
Healthcare workers	34 (72)

### Relative analysis (age and gender).

**FIG 4:**
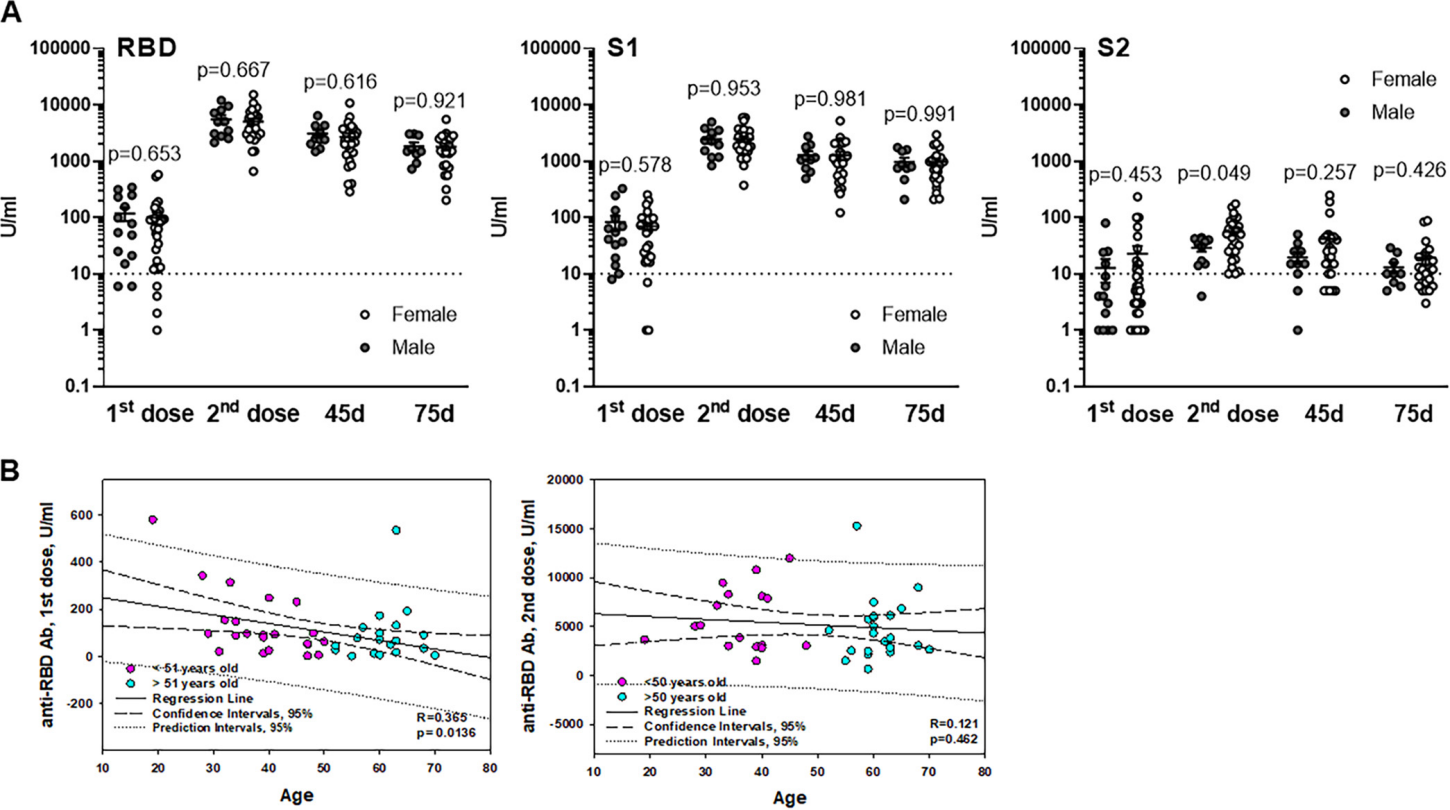
Gender and age differences in antibody responses to mRNA vaccination. Antibody responses to two doses of Pfizer and Moderna vaccinations of healthy volunteers were assessed by the BioPlex 2200 SARS-CoV-2 IgG multiplex assay. (A) Results represent individual values (dots) and means ± the SEM of IgG antibodies recognizing RBD, S1, and S2 SARS-CoV-2 antigens in males and females 2 weeks after the first and second doses and 45 and 75 days after the second shot. The *y* axis represents antibody levels in U/ml in logarithmic scale. *P* values were calculated using an unpaired *t* test. (B) Pearson correlation analysis of antibody responses versus age of healthy volunteers. Two age cohorts separated based on the median age (50) are shown for clarity. Induction of anti-RBD antibody after first (left panel) and second (right panel) doses of a vaccine are shown in the *y* axis. Regression lines and correlation coefficients with the corresponding *P* values are shown. CIs (95%) and prediction intervals (95%) are also indicated.

We next evaluated whether the antibody response to mRNA vaccination was similar in males and females. Comparative results are shown in Fig. 4A. An unpaired *t* test analysis revealed no statistically significant differences between vaccinated males and females in anti-RBD and anti-S1 antibody levels after the first and second doses and at the later time points, that is, 45 and 75 days after the second shot. However, a statistically significant difference between males (28.91 ± 4.19 U/ml) and females (55.93 ± 8.11 U/ml) in anti-S2 response after the second dose was demonstrated (Fig. 4A). Although at 45 days after the second injection the levels of anti-S2 antibodies were higher in females (41.31 ± 11.44 U/ml) than in males (19.60 ± 4.64 U/ml), these differences did not reach statistical significance. Thirty days later, this difference was minimal: ∼19 U/ml versus ∼13 U/ml. Thus, we did not observe gender-associated differences in a long-term response to mRNA vaccination.

Next, we sought to determine whether specific antibody responses to mRNA administration may be age dependent within adults aged 19 to 70. Figure 4B shows antibody responses versus age separated based on the median age (51 years old). Pearson correlation analysis utilizing all age groups revealed a statistically significant relationship between the age and level of anti-RBD IgG after first dose of vaccine administration (Fig. 4B). However, no age dependency of the antibody response was seen after the second dose, suggesting that immune boosting was significant in all age categories. No correlations between the anti-RBD antibody levels and age were detected at later time points of blood collection (45 and 75 days after second dose administration). Similarly, induction of anti-S1 IgG after first vaccine dose was age dependent (*R* = 0.410, *P* = 0.005), whereas this correlation disappeared after second dose (*R* = 0.234, *P* = 0.152) and was not detected at later time points (*R* < 0.02, *P* > 0.9). No significant correlations between age and anti-S2 response were determined (data not shown). Thus, induction of anti-RBD and anti-S2 antibodies by vaccination was initially inversely correlated with volunteers’ age, while the later response and its duration were age independent.

### Comparative examples (COVID-19, vaccination interval, and vaccination after a natural disease).

**FIG 5:**
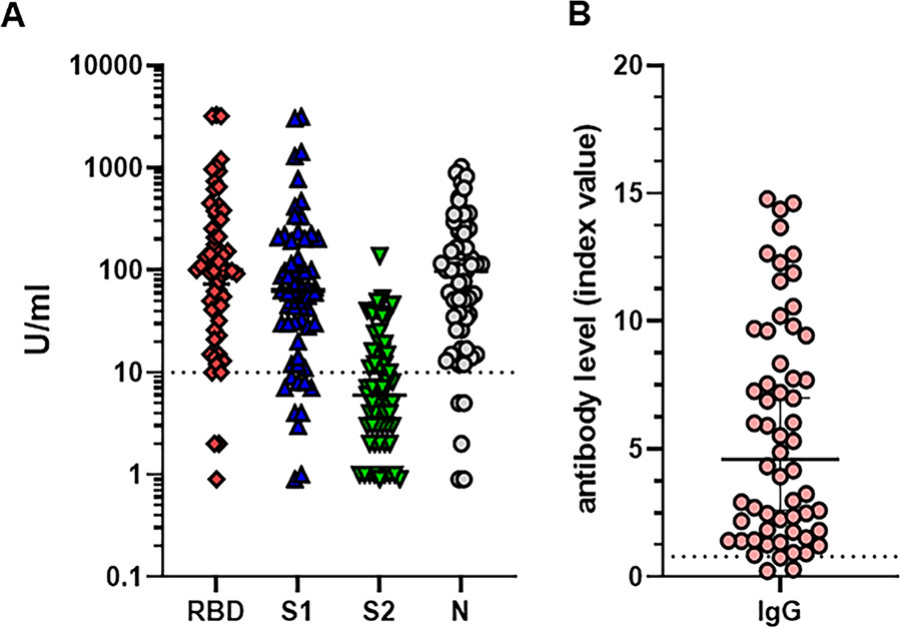
Serum anti-SARS-CoV-2 antibody levels in COVID-19 recovered individuals. Antibody levels were assessed by using the BioPlex 2200 SARS-CoV-2 IgG multiplex panel (A) and the EuroImmun IgG ELISA (B) in 59 COVID-19-recovered persons 3 to 8 months after disease. Antibody levels are expressed in U/ml in logarithmic scale (A) and index values (B), as described in Materials and Methods. 10 U/ml (dotted line) is the negative cutoff concentration for panel A. A, index value of 1 (dotted line) is the negative cutoff concentration for panel B. Results from a representative cohort of patients are shown.

**FIG 6:**
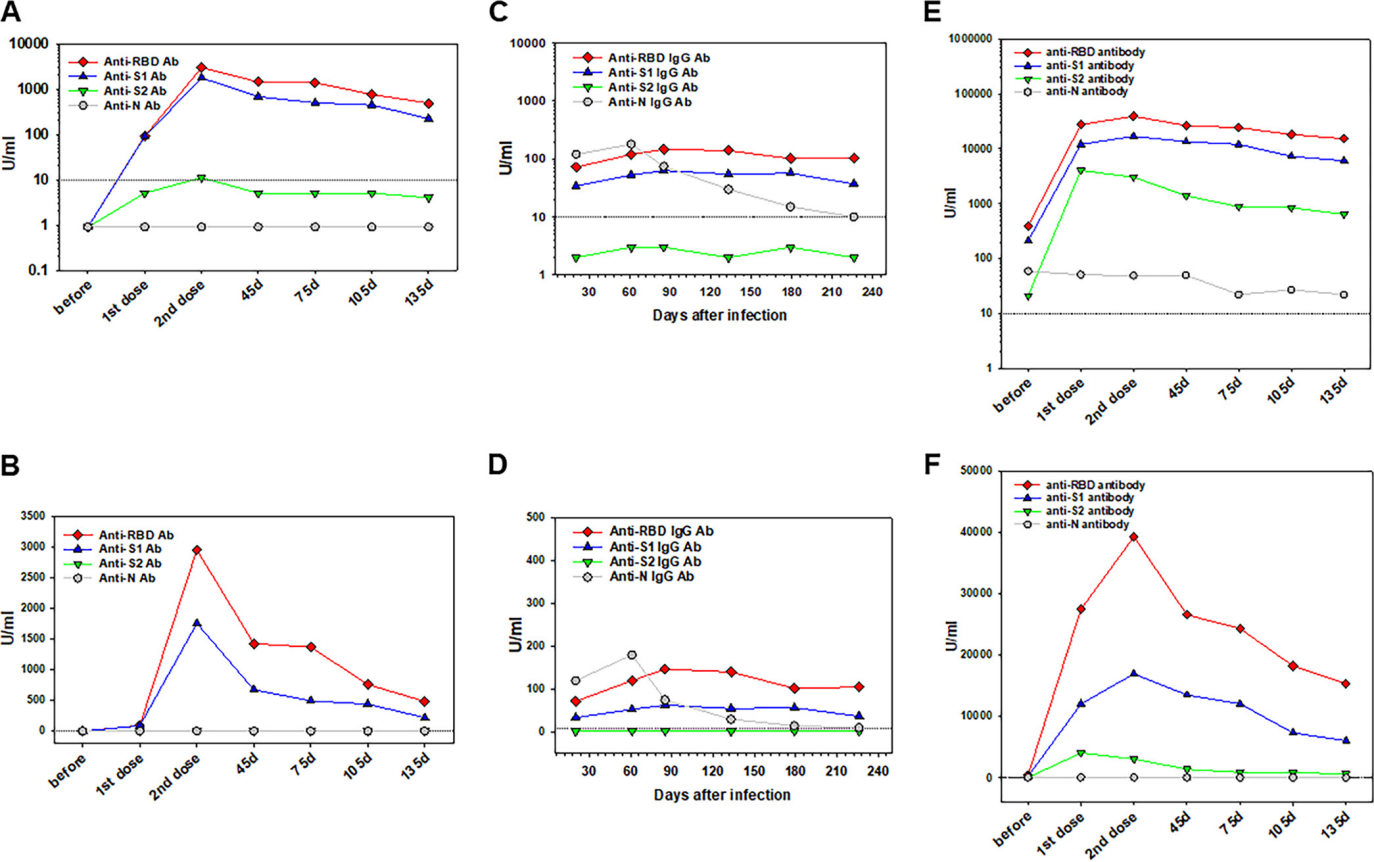
Vignettes to compare healthy to previously infected volunteers. Examples of anti-SARS-CoV-2 antibody levels in a vaccinated healthy volunteer (A and B), a mild COVID-19 recovered person (C and D), and a previously SARS-CoV-2-infected individual receiving a two-dose mRNA vaccine (E and F) are shown. Antibody levels were assessed by using a BioPlex 2200 SARS-CoV-2 IgG multiplex panel. Results are expressed as antibody levels in U/ml (*y* axis) in the linear (B, D, and F) and logarithmic (A, C, and E) scales for comparison. The *x* axis indicates time points before, 2 weeks after the first and second vaccine doses, and 45, 75, and 105 days after the second shot (A, B, E, and F) or days after natural disease (C and D). 10 U/ml (dotted line) is the negative cutoff concentration.

For comparative presentation of anti-SARS-CoV-2 serum antibody levels determined by a multiplexed assay, we determined the antibody response in randomly selected samples obtained from COVID-19-confirmed and COVID-19-recovered patients who donated their blood for convalescent plasma evaluation. A comprehensive analysis of antibody levels in different cohorts of COVID-19 patients have been recently reported by our lab (N. Cook et al., unpublished data). Figure 5A demonstrates a range of anti-SARS-CoV-2 antibodies in individuals recovered (3 to 6 months) after mild COVID-19 incidence. The levels of tested antibodies were similar to the levels of antibodies seen in individuals receiving a first dose of COVID-19 mRNA vaccines, with the only difference being the absence of anti-nucleocapsid antibody in vaccinated healthy volunteers (Fig. 6). As an additional confirmation of positive anti-SARS-CoV-2 immunity in COVID-19-recovered patients, we utilized the EuroImmun assay detecting anti-S IgG antibodies in the same blood specimens (Fig. 5B). Together, these results suggest that anti-N antibody may serve as a marker of previous COVID-19 disease and that two-dose mRNA vaccination induces a significantly higher antibody response than that seen in people recovered after mild or medium SARS-CoV-2 infection.

A few examples from participating volunteers open additional opportunities for further studies. Figure 6 demonstrates individual results for comparison: a representative antibody response to a two-dose vaccination in a volunteer in a linear (Fig. 6B) and a logarithmic (Fig. 6A) scale. The peak of antibody response to RBD antigen reached ∼3,000 U/ml. The level of anti-SARS-CoV-2 antibodies in an individual recovered after mild COVID-19 was within 50 to 150 U/ml during ∼8 months after a disease, with a slight but consistent declining of anti-RBD and anti-S1 antibodies and a marked reduction of anti-N antibody (Fig. 6): the results are shown in a linear (Fig. 6D) and a logarithmic (Fig. 6C) scale. This suggests that vaccine-induced antibodies are seen at higher levels compared to mild natural disease-induced antibodies at least during a few months after vaccination. Interestingly, vaccination of a person several months after mild confirmed COVID-19 recovery resulted in a dramatic induction of anti-RBD, -S1, and -S2, but not anti-N, antibodies up to 40,000 U/ml, which is almost 1,000-fold higher than in healthy vaccinated volunteers (Fig. 6E and F). Importantly, although the second vaccine dose resulted in the highest boosting of antibody production, even the first dose stimulated a dramatic antibody response in a COVID-19-recovered person, which was significantly higher than the average response in previously COVID-19-free volunteers. Together, these results of the multiplexed analysis of anti-SARS-CoV2 antibodies support the idea that both anti-N and anti-S2 antibodies may be helpful in differentiation of individuals who have had a previous infection for at least several months earlier and were or were not vaccinated.

**FIG 7:**
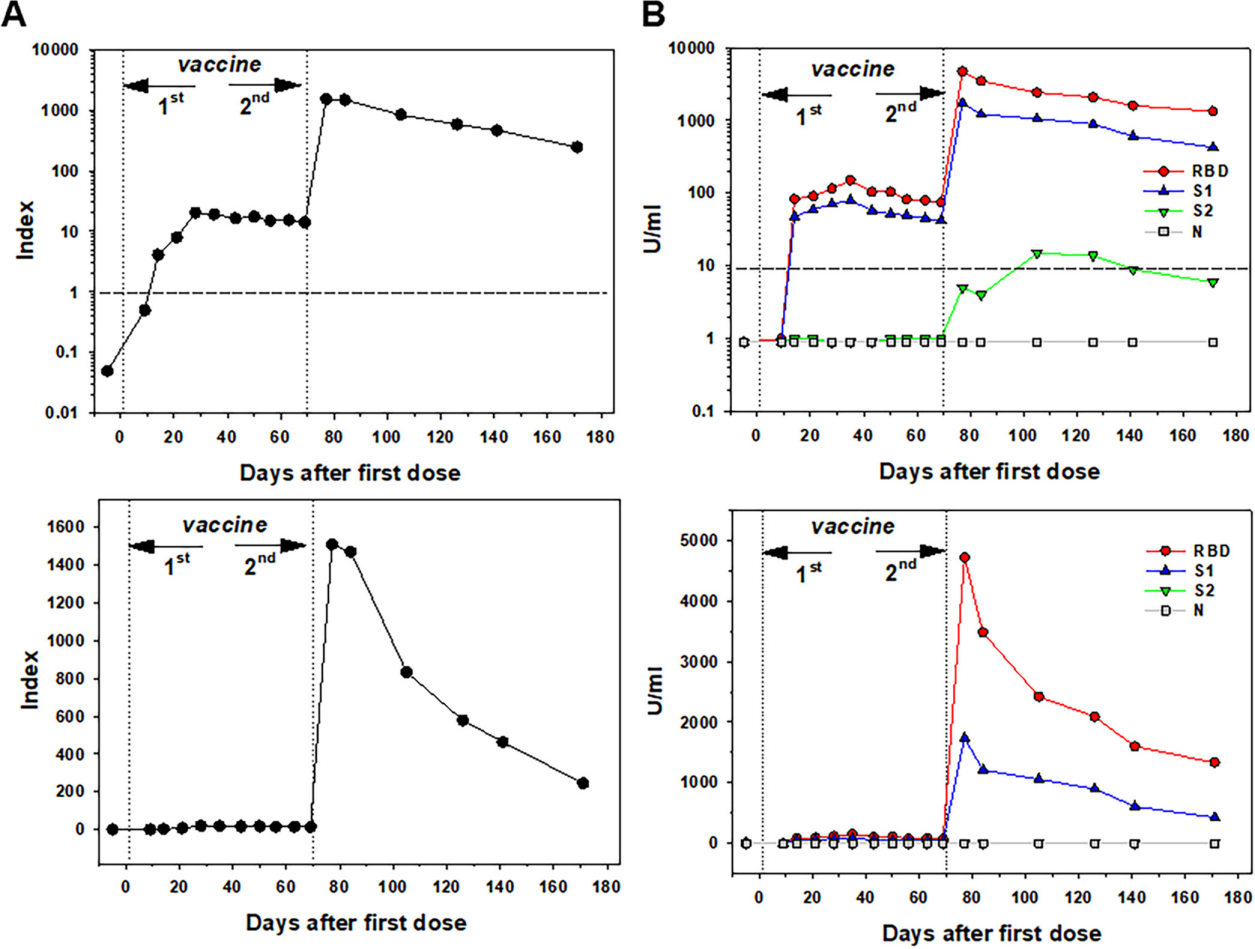
Anti-SARS-CoV-2 antibody levels in a vaccinated healthy volunteer with a delayed boosting vaccination. Antibody levels were assessed by using the Siemens SARS-CoV-2 total assay (A) and the BioPlex 2200 SARS-CoV-2 IgG multiplex panel (B). The results are shown with linear (lower panels) and logarithmic (upper panels) scales (*y* axis) in index values (A) and U/ml (B). The *x* axis indicates the days after the first dose of a vaccine administration. An index unit of 1 (dotted line) is the negative cutoff concentration for panel A. 10 U/ml (dotted line) is the negative cutoff concentration for panel B.

Figure 7 shows prolonged serum antibody levels in a healthy volunteer after the first dose of the Moderna vaccine for up to 70 days prior to administration of the second dose of the vaccine. As expected, a relatively low antibody response, determined by the BioPlex SARS-CoV-2 IgG assay, was seen after first injection (Fig. 7B) to both RBD and S1 antigens, as shown in the linear (lower panel) and logarithmic (upper panel) scales. Antibody levels slowly and gradually decreased for 70 days, while a boosting vaccination induced 30- to 40-fold increase in antibody levels, peaking 1 week after injection. The following decline in serum antibodies was also anticipated. These results were verified by determining antibodies utilizing Siemens SARS-CoV-2 total assay (Fig. 7A). The results, shown linear (lower panel) and logarithmic (upper panel) scales, confirmed a persistent low level of specific antibodies after the first dose and a significant boosting induced by the second dose of a vaccine. Together, these data demonstrate that waiting longer between the two doses could achieve the same effect in healthy individuals.

In summary, our results demonstrate clinical usefulness of multiplexed detection of anti-SARS-CoV-2 antibodies in vaccinated healthy volunteers. Specifically, we revealed a compatible efficacy of the Pfizer-BioNTech and Moderna vaccines in the induction of various anti-SARS-CoV-2 antibodies, a similar immune response to vaccination in males and females, an initial dose-limited age dependence of antibody production, and a marked monthly decline in antibody levels after vaccination.

## DISCUSSION

Currently, there are three basic types of tests to determine whether an individual has been infected with SARS-CoV-2: viral RNA detection, viral antigen detection, and detection of antibodies to the virus. Viral tests are used to assess acute infection, whereas antibody tests provide evidence of prior infection. Although the FDA has not authorized the use of antibody tests for the diagnosis of acute infection, they are the key tests for assessing immune response to a COVID-19 vaccination, distinguishing immune response to a vaccine or natural disease and identification of prior asymptomatic infections.

There are four major structural proteins in SARS-CoV-2: spike, envelope, membrane, and nucleocapsid encoded by the S, E, M, and N genes, as well as nonstructural proteins (13, 28). The S protein contains the S1 and S2 subunits which mediate receptor binding and membrane fusion, respectively. Viral entry is achieved through virus-host cell membrane fusion, which requires a conformational change in the S protein. The RBD in S1 binds to angiotensin-converting enzyme 2 (ACE2) on the host cell, which initiates the necessary conformational change in the S2 subunit that allows for membrane fusion (28). Both antibody and T cell responses are detectable to all major viral antigens during or after COVID-19 (29, 30). Similar immune responses can be seen after COVID-19 vaccination. Although more than 200 vaccine candidates are in development, of which more than 60 are in clinical development, as of May 2021 the WHO has determined that the following vaccines against COVID-19 have met the necessary criteria for safety and efficacy: vaccines made by SinoPharm, AstraZeneca/Oxford, Johnson and Johnson, Moderna, and Pfizer-BioNTech. Some national regulators have also assessed other COVID-19 vaccine products for use in their countries. All major first-generation COVID-19 vaccines approved in the United States utilize spike protein coding for immune response targeting and thus induce RBD, S1, and S2 antibody production. Interestingly, the Pfizer phase I/II COVID-19 vaccine trial with BNT162b1, an mRNA that encodes the RBD of the spike protein, showed that a detectable level of RBD-binding IgG was seen before the second dose, but the peak was 1 week after the booster (31).

For Moderna, there were detectable antibodies by the day of second dose with the peak on day 15 after second injection (14). The follow-up data for the Moderna mRNA-1273 vaccine provide immunogenicity data set 90 days after the second vaccination, showing high levels of RBD binding and neutralizing antibodies that declined slightly over time, as expected (32). Similarly, blood from six adults receiving the mRNA-based SARS-CoV-2 vaccines was analyzed for the immune responses to S protein and RBD by ELISA (16). This study found that immune responses were highest 1 week after the second dose of vaccine with a subsequent decline in antibody titers. Neutralizing antibody titers displayed a comparable trend for all vaccinees (16). These results were confirmed by retrospective analysis of healthy donors’ serologic response to immunization with the Pfizer BNT162b2 vaccine. All participants demonstrated no S protein (RBD) antibodies before vaccination and were all positive for S protein antibodies by 2 weeks after the first vaccine dose. The serum levels of S protein antibodies peaked at 4 to 5 weeks following the initial vaccine dose (33). Though correlates of protection against SARS-CoV-2 infection are not yet widely proven, our results and those of others signify that even with a slight and expected decrease in the level of circulating binding and neutralizing antibodies, mRNA vaccines have the ability to deliver lasting humoral immunity.

Utilizing a new multiplexed antibody detection approach here, we demonstrated induction of diverse anti-spike antibodies after both the first and the second doses of spike protein encoding Moderna and Pfizer vaccines, including a strong response to RBD, which is the major target of neutralizing antibodies in convalescent patients (34). However, some isolated neutralizing antibodies may exert their function without interfering with RBD-ACE2 recognition or even without binding to RBD (35–37). Indeed, while RBD-binding antibodies are commonly referred to as “neutralizing” antibodies, there is not a full correlation between the level of RBD-binding antibodies and the real neutralizing potential of plasma or serum. For example, it was reported that the concordance between seven different anti-SARS-CoV-2 immunoassays and virus neutralization tests varied widely (38). Although these observations may suggest that neutralization potency, as opposed to antibody to epitope specificity, is responsible for the putative protection of anti-SARS-CoV-2 antibodies (39), these results also advocate that detection and characterization of immune response to multiple viral protein domains and antigens is well justified.

In our study, the anti-RBD response was the strongest, followed by the anti-S1 response, whereas the anti-S2 response was the lowest in all tested individuals. While RBD and S1 antibodies are commonly associated with their ability to neutralize a virus, the clinical significance of antibodies recognizing the S2 subunit is less clear. Recently, Voss et al. reported that more than 80% of the anti-spike IgG repertoire bound to epitopes outside the RBD, with about 40% of the circulating antibodies targeting the S2 subunit (40). Protective activity of the neutralizing anti-S2 antibody has been also reported (35, 39, 41). Thus, our data demonstrate that tested vaccines, in addition to the anti-RBD and anti-S1 responses, are also eliciting low but significant antibodies targeting the S2 subunit, which are likely providing another layer of protection against the virus. Interestingly, recent characterization of binding and neutralizing antibodies isolated from SARS-CoV-2-infected subjects revealed that the anti-S2 antibody was the only one that was unaffected by mutations found in the recently emerged South African variant (39). The authors of that study concluded that these antibodies can serve as blueprints for the development of immunogens to elicit protective neutralizing antibody responses against multiple coronaviruses. Our findings suggest that these vaccines elicit polyclonal responses against multiple epitopes, which may offer enhance protection against variants.

In our study, no differences between Pfizer-induced and Moderna-induced antibody responses were detected (Fig. 3). This agrees with other work in a study of a cohort of 20 volunteers who received either the Moderna or the Pfizer/BioNTech vaccines. Wang et al. demonstrated high levels of IgM and IgG anti-SARS-CoV-2 spike protein and RBD binding titers 8 weeks after the second vaccine injection (18).

We also did not detect significant differences in immune response between males and females but demonstrated that the initial response to different antigens was age dependent. However, this correlation was observed only during the relatively weak response to the first dose and disappeared after boosting of the immune reaction with a second dose (Fig. 4). These results have been recently confirmed (23). The results of another study, analyzing the antibody titer 7 days after the second dose of Pfizer BNT162b2 vaccine in health care workers, revealed that females, lean people, and young people have an increased capacity to mount humoral immune responses compared to male, overweight, and older populations (42). Although these data confirm the age dependence of vaccine-induced humoral immune response detected in our study, we did not see a significant gender-dependent responses in our cohort of healthy volunteers. Importantly, our study includes only IgG antibody evaluation. Recent data revealed that in response to BNT162b2, volunteers developed only moderate levels of anti-S1 IgA and IgM antibodies after both the first and the second doses of the vaccine, in contrast to the strong IgG response (23). Similarly, the BNT162b2 vaccine compared to natural infection induces low anti-S and anti-RBD IgM and IgA responses but does induce a strong IgG response (43). One possible explanation for the relatively low IgM and IgA responses to the vaccine are the lipid components of the vaccine formulation, which are relatively uncharacterized with respect to their effect on the human immune system. Some early work indicates that the lipid components may increase Th1-polarized CD4^+^ T cell responses, thus creating early IgG class-switching that could produce the observed high IgG and low IgA and IgM responses (43). However, Wang et al. reported high levels of IgM and IgG anti-S and anti-RBD binding titers in volunteers 8 weeks after the second vaccine injection (18). Therefore, additional studies of different cohorts of vaccinated people are needed in order to draw conclusions regarding the significance of polarized antibody responses after COVID-19 vaccination.

Our data demonstrating a strong antibody response in an individual who experience a natural disease 9 months earlier (Fig. 6C) are in agreement with the results demonstrating that the antibody response to the first vaccine dose in individuals with preexisting immunity is equal to or even exceeds the titers found in naive individuals after the second dose (17, 44). These authors studied more than 100 vaccine recipients, ∼40% of whom were seropositive for SARS-CoV-2 at the time of vaccination. They found that median antibody titers among seropositive vaccinees after the first dose were more than 10 times higher than titers among seronegative vaccinees after the second dose. The fact that after a single dose of COVID-19 vaccine, people with a prior COVID-19 infection had antibody levels similar to those of people without prior infection after two vaccine doses suggests the importance of testing anti-SARS-CoV-2 antibodies prior to vaccination in order to prevent immune overboosting and limit potential adverse effects, as well as to minimize the unnecessary utilization of vaccines in countries with restricted vaccine availability.

In conclusion, our results suggest that postvaccination testing of multiple antibody responses is a vital and practicable instrument for following vaccinated people for selecting individuals who need additional boosting because of low responsiveness or might require a third dose of vaccine at an earlier time point or persons who may do not need second dose due to previous SARS-CoV-2 infection. For instance, measurement of SARS-CoV-2 IgG production in patients with hematological malignancy who received two mRNA vaccine doses revealed that 46% of these patients did not produce antibodies and were therefore vaccine nonresponders. Patients with B-cell CLL were at a particularly high risk, with only 23% having a detectable antibody response even though almost 70% of the B-cell CLL patients were not concurrently receiving cancer therapy (45). Alternatively, if a cohort of presumably healthy individuals demonstrates an unexpectedly low response to vaccination, concerns can be raised regarding a particular batch of a vaccine. Our and other studies characterizing the clinically testable response to COVID-19 vaccination pave the way toward developing effective tools to combat the pandemic.

## MATERIALS AND METHODS

### Study design and participants.

The goal of this prospective panel study was to examine healthy individuals receiving the Pfizer/BioNTech and Moderna COVID-19 vaccines to characterize the serum levels of antibodies recognizing four virus antigens before and at different time points after the first and second doses of vaccines. Eligible participants were healthy individuals aged 19 or older. A total of 47 volunteers who received two injections of either vaccine 3 or 4 weeks apart (Pfizer/BioNTech and Moderna, respectively) participated in the study (Table 1). The key exclusion criteria included an axillary temperature of more than 37°C, a history of allergy to any vaccine components, anemia and treatment for anemia or iron deficiency, and immunosuppression whether from disease or treatment. Written informed consent was obtained from each participant before enrollment. As a control (natural disease), remnant samples from patients who had SARS-CoV-2 antibody ordered as part of their clinical care or for convalescent plasma donation were utilized. Written approval of the study protocol and informed consent form were obtained from the relevant Independent Ethics Committee/Institutional Review Board (IRB) of the University of Pittsburgh (studies 20040072 and 20120157). The study was conducted in compliance with Good Clinical Practice and all applicable laws and guidelines consistent with ethical principles of the Declaration of Helsinki.

Blood samples were collected 6 to 72 h prior to the initial vaccine administration, 2 weeks (14 to 16 days) after the first dose of either vaccine, 2 weeks after the second dose, and then monthly at day 45 (42 to 46 days), day 75 (73 to 78 days), 105 (102 to 108 days), and 135 (132 to 138) after the completed vaccination.

### Detection of virus-specific antibodies.

Blood samples were collected into BD serum gel separator tubes and centrifuged after complete clotting at room temperature (1,200 × *g*, 10 min). All specimens were deidentified and aliquoted for assessment. Serum samples were stored at −30°C for 1 to 3 months before analysis.

SARS-CoV-2 antibody assays were performed in CLIA certified high-complexity clinical laboratories at the (University of Pittsburgh Medical Center). For screening and differentiation of the antibody response to COVID-19 vaccines, we used the BioPlex 2200 CoV-2 IgG Panel (Bio-Rad Laboratories, Inc., Hercules, CA). The BioPlex 2200 SARS-CoV-2 IgG panel is a multiplex assay for the qualitative (IgG screen) and semiquantitative (U/ml) detection of IgG class antibodies against the RBD, S1, S2, and nucleocapsid (N) proteins of the SARS-CoV-2 virus in human serum and plasma. The analytical measuring range was 1 to 100 U/ml, with onboard dilutions of 1:8, 1:16, and 1:32. Samples that displayed any antibody levels higher than 3,200 U/ml were manually diluted 1:5 or 1:10 using the Bio-Rad dilution solution. The SARS-CoV-2 IgG calibrator set included five levels of each antibody specificity, with a 4PL calibration curve fit; for the SARS-CoV-2 IgG quality control, two levels were utilized. Performance studies showed an overall specificity of 99.8% and an overall sensitivity of 96.3%, according to the manufacturer’s instructions for use. Evaluation and validation of this assay in our lab confirmed these characteristics (Cook et al., unpublished). Results that are <10 U/ml are considered negative, and positivity is ≥10 U/ml.

For the expansion and verification of serum antibody results, we also used an ADVIA Centaur SARS-CoV-2 total (COV2T) chemiluminescent immunoassay (Siemens USA, Malvern, PA) intended for qualitative detection of total antibodies, including IgG and IgM, to SARS-CoV-2. The COV2T assay on ADVIA Centaur XP system is a fully automated one-step antigen sandwich immunoassay using acridinium ester chemiluminescent technology, in which antigens are bridged by antibodies present in the patient sample. The solid phase contains a preformed complex of streptavidin-coated microparticles and biotinylated SARS-CoV-2 recombinant S1-RBD antigen. A direct relationship exists between the level of SARS-CoV-2 antibodies present in the patient sample and the amount of relative light units (RLUs) detected by the system. A result of reactive or nonreactive is determined according to the index value established with the high and low calibrators. Within the measuring interval 0.05 to 10.00 index, results were reported as nonreactive (<1.0 index) or reactive (≥1.0 index). Manual dilution of serum samples (1:10 to 1:1,000) was utilized if required. This assay was evaluated in the lab and the results were published (46).

For additional confirmation of antibody results, we used an anti-SARS-CoV-2 ELISA (EuroImmun, NJ) that provides semiquantitative determination of human IgG antibodies targeting spike protein, which has been evaluated and verified in our lab previously (46, 47). The assay was run according to the manufacturer provided protocol using an iMark microplate absorbance reader (Bio-Rad Laboratories). The absorbance of sample wells was measured immediately at 450 and 630 nm, with output reports generated with the optical density (OD) at 630 nm subtracted from the OD at 450 nm. Data were then analyzed as recommended by the manufacturer as a ratio based on the sample OD divided by the averaged OD of the calibrators. This ratio was interpreted as follows: <0.8, negative; ≥0.8 to <1.0, borderline; and ≥1.1, positive. Antibody level results are expressed as the “index value.”

### Statistical analysis.

First, data were compared using *t* tests on nontransformed and log-transformed values. Evolution of antibody levels over time was assessed using paired *t* tests. For a single comparison of two groups, a Student *t* test was used after evaluation of normality. If the data distribution was not normal (Kolmogorov-Smirnov normality test), a Mann-Whitney rank sum test was performed. To compare multiple groups, ANOVA was applied. We used ANOVA to compare log-transformed and nontransformed antibody levels. When comparison of all groups showed a significant difference, we performed pairwise comparisons. Correlation between tested parameters was done using Pearson correlation. Hypothesis testing was two-sided, and we considered *P* values of <0.05 to be significant.

To analyze variability and central tendencies and summarize immune response profiles after vaccination, reverse cumulative distribution curves (RCDCs) were generated. These graphic techniques were used by Salk to display distribution of anti-polio antibodies (48). The method was further developed by Reed et al. to analyze antibody response in vaccine studies (49). RCDCs are step functions based on the order statistics of the data. The curves begin with a value of 1.0 or 100% at an antibody titer of virtually zero and fall to a value of zero above the largest titer value in steps of 1/*n*. If there are ties, then the step size is equal to the number of tied values times 1/*n* (50). We calculated antibody levels and corresponding 95% confidence intervals (CIs) on the basis of standard normal distribution of the log-transformed antibody concentrations.

We used SigmaPlot (version 14; Systat Software, Inc., San Jose, CA) and Prism (version 9; GraphPad, San Diego, CA) for all analyses and data presentation. The data are presented as means ± the standard errors of the mean (SEM) or medians with a 95% confidence interval (CI), as stated in the corresponding figure legends or in the text.
